# The effect of salicylic and jasmonic acids on the activity of SnAGO genes in the fungus Stagonospora nodorum Berk. in in vitro culture and during infection of wheat plants

**DOI:** 10.18699/VJGB-23-115

**Published:** 2023-12

**Authors:** M.Yu. Shein, G.F. Burkhanova, I.V. Maksimov

**Affiliations:** Institute of Biochemistry and Genetics – Subdivision of the Ufa Federal Research Centre of the Russian Academy of Sciences, Ufa, Russia; Institute of Biochemistry and Genetics – Subdivision of the Ufa Federal Research Centre of the Russian Academy of Sciences, Ufa, Russia; Institute of Biochemistry and Genetics – Subdivision of the Ufa Federal Research Centre of the Russian Academy of Sciences, Ufa, Russia

**Keywords:** RNA interference, SnAGO genes, fungus Stagonospora nodorum, common wheat, pathogenesis, salicylic acid, jasmonic acid, РНК-интерференция, гены SnAGO, гриб Stagonospora nodorum, мягкая пшеница, патогенез, салициловая кислота, жасмоновая кислота

## Abstract

RNA interference is a gene silencing mechanism that plays an important role in genetic regulation in a number of eukaryotes. Argonaute (AGO) proteins are central to the complex RNA interference system. However, their role in this mechanism, both in the host plant organism and in the pathogen, has not yet been fully elucidated. In this work, we identified and phylogenetically analyzed the SnAGO1, SnAGO2, SnAGO3, and SnAGO18 genes of the pathogenic fungus Stagonospora nodorum Berk., and analyzed their expression under conditions of infection of plants with varying degrees of resistance to the pathogen. The expression level against the background of plant immunization with the resistance inducers salicylic and jasmonic acids was assessed. In addition, the activity of these genes in the culture of the fungus in vitro was studied under the direct influence of resistance inducers on the mycelium of the fungus. Earlier activation of the SnAGO genes in in vitro culture under the influence of salicylic and jasmonic acids suggests their sensitivity to it. In an in vivo system, plant immunization to induce the accumulation of pathogen SnAGO transcripts was found. At the same time, the SnAGO genes of the fungus S. nodorum, when interacting with plant cells, reacted depending on the degree of host resistance: the highest level of transcripts in the resistant variety was observed. Thus, our data prove that the SnAGO genes of the fungus S. nodorum effectively interact with the host defense system in direct proportion to the degree of resistance of the latter to the pathogen. It was proposed to use the ratio of the transcriptional activity of the fungal reference gene SnTub to the host TaRLI gene as a marker of disease development in the initial period of the infectious process.

## Abstract (ru)

РНК-интерференция представляет собой механизм подавления генов, играющий важную роль в генетической регуляции у эукариот. Белки Argonaute (AGO) занимают центральное место в сложной системе явления РНК-интерференции. Однако их роль в этом механизме, как в организме растения-хозяина, так и у патогена, до сих пор полностью не исследована. Мы провели идентификацию и филогенетический анализ генов SnAGO1, SnAGO2, SnAGO3 и SnAGO18 патогенного гриба Stagonospora nodorum Berk., возбудителя септориоза пшеницы, и проанализировали их экспрессию в условиях инфицирования растений с различной степенью устойчивости к патогену. Уровень экспрессии оценивали на фоне иммунизации растений индукторами устойчивости: салициловой и жасмоновой кислотами. Также изучена активность указанных генов в культуре гриба при непосредственном воздействии индукторов устойчивости на мицелий гриба. Выявленная более ранняя активация генов SnAGO в культуре под влиянием салициловой и жасмоновой кислот указывает на их чувствительность к ним. В системе in vivo обнаружено, что иммунизация растений индуцирует накопление транскриптов SnAGO патогена. При этом гены SnAGO гриба S. nodorum при взаимодействии с растительными клетками реагировали в зависимости от степени устойчивости хозяина: наиболее высокий уровень транскриптов наблюдался в устойчивом сорте. Таким образом, полученные данные доказывают, что гены SnAGO гриба S. nodorum эффективно взаимодействуют с системой защиты хозяина в прямой зависимости от степени устойчивости последнего к патогену. Предложено использовать отношение транскрипционной активности грибного референсного гена SnTub к хозяйскому гену TaRLI в качестве маркера развития болезни в начальный период инфекционного процесса.

## Introduction

Phytopathogenic fungi are a powerful risk to food security, which limits the biological potential of agricultural plants and reduces the quality of the resulting products. At the present stage, methods of plant protection are being developed based on natural systemic and cellular phytoimmunity, where a special place is occupied by a unique mechanism that disables gene expression, described by the term “RNA interference” (RNAi) – an evolutionarily conservative and at the same time highly specific immune component for almost all eukaryotes.

Methodologically, when creating modern approaches to plant protection, it is necessary to take into account that during the interaction of plants with pathogens, especially fungal ones, the RNAi components of not only the host, but also the pathogen are activated. Induction of the activity of a number of genes responsible for the functioning of RNAi in pathogenic fungi suggests the possibility of their participation in the suppression of genes of the host defense system (Weiberg et al., 2013). It should be noted that the role of the RNAi mechanism in the evolution and life of fungi is important and can vary quite significantly depending on the survival strategy, the method of infection and spread, as well as the pathogens that infect the fungus itself (Neupane et al., 2019). For example, it is known that artificial inactivation of one or two genes encoding RNAi proteins in phytopathogenic fungi disrupts virulence (Raman et al., 2017; Wang et al., 2018).

Argonaute proteins (AGOs) are considered key components in the complex RNAi phenomenon and bind short microRNAs (Feng et al., 2017; Neupane et al., 2019). The most important function of AGO proteins, actively discussed in the scientific literature, is participation in phytoimmunity. For example, we previously observed that pre-treatment of seeds with salicylic acid (SA) formed the resistance of wheat to Septoria nodorum blotch (SNB), and at the same time, active accumulation of TaAGO1 gene transcripts was observed in plant tissues infected with the causative agent of this disease (Shein et al., 2021). So, in tobacco plants Nicotiana attenuata Torr. ex S.Watson, the accumulation of the NaAGO4 protein turned out to be critical in the formation of resistance to the fungus Fusarium brachygibbosum Padwick (1945) through the jasmonate signaling pathway (Pradhan et al., 2020). Disruption of this process turned off the synthesis of jasmonic acid (JA) in plants and led to their infection, but resistance to the fungus was restored after treating the plants with JA. It can be assumed that the functioning of the plant defense system, regulated by JA, mediates the operation of the mechanism of the RNAi phenomenon. The importance of plant proteins AGO18 in the formation of antiviral defense in rice was discovered (Yang et al., 2020).

It is also known that AGO proteins are actively involved in physiological processes occurring in the mycelium of various types of fungi. Thus, genes encoding AGO proteins were identified in the genome of the fungi Fusarium graminearum (FgAGO1) (Chen et al., 2015) and Metarhizium robertsii (MrAGO1) (Meng et al., 2017). The participation of fungal proteins of the AGO family in the formation of compatibility between the host and the pathogen was demonstrated in the fungi Verticillium dahlia and V. longisporum (Shen et al., 2014). A similar effect was observed in the fungus Scletotinia sclerotiorum: mutants of the AGO2 gene of this fungus had slow growth and reduced virulence (Neupane et al., 2019). Suppression of AGO2 (QDE-2) gene expression also reduced virulence in the fungi Valsa mali (Feng et al., 2017) and Fusarium oxysporum f. sp. lycopersici (Jo et al., 2018).

Thus, proteins of the AGO family are key components in the functioning of not only plant but also fungal defense systems mediated by RNAi mechanisms. At the same time, it should be noted that so far little work has been devoted to the analysis of the expression of genes encoding proteins of the RNAi mechanism in various pathogens under conditions of plant infection and preliminary immunization with phytohormones SA and JA. In this work, such an analysis was carried out using a model of the phytopathogenic fungus Stagonospora nodorum Berk. (Septoria, Parastagonospora, Phaeosphaeria), which causes SNB – one of the most harmful wheat diseases. The main objective was to evaluate changes in the transcriptional activity of the SNAGO genes encoding AGO proteins in an in vitro culture of the fungus and under conditions of infection of plants with this fungus, contrasting in resistance to SNB against the background of SA and JA treatment.

## Materials and methods

Object. In the experiment, we used a highly virulent strain of the phytopathogenic fungus S. nodorum SnB against common wheat Triticum aestivum L. (Zhnitsa cultivar) from the collection of the Institute of Biochemistry and Genetics – Subdivision of the Ufa Federal Research Centre of the Russian Academy of Sciences. As plant material, we used two cultivars of spring bread wheat T. aestivum (BAD, 2n = 42) contrasting in their resistance to SNB: Zhnitsa (susceptible) and Omskaya 35 (resistant).

Cultivation of the fungus in vitro. The fungus was cultivated on a liquid potato-glucose nutrient medium in Petri dishes. To do this, a suspension of fungal spores was added to the nutrient medium at a rate of 105 spores/ml and cultivated in a KBW E6 climate chamber (Binder GmbH, Germany) at a temperature of 18 °C for 14 days with periodic 16-hour lighting. Solutions of SA with concentrations of 10–4, 10–5 and 10–6 M, as well as JA solutions with concentrations of 10–5, 10–6 and 10–7 M were previously added to the experimental versions of the nutrient media. These concentrations were chosen by us as optimal due to the data previously obtained in our laboratory on the influence of SA and JA on the degree of development of SNB in various wheat cultivars in the pathosystem (Yarullina et al., 2011).

The design of the experiment. Wheat seeds were presoaked for 24 hours in solutions containing 10–5 M SA and 10–7 M JA, or their composition. Control samples were kept in distilled water for the same period of time. Then, the seedlings in isolated vessels on Hoagland–Arnon nutrient medium were placed in a KBW E6 plant growth chamber (Binder GmbH, Germany), with a 16-hour photoperiod at a temperature of 20/24 ºC (night/day). The noted concentrations of SA and JA were selected as a result of preliminary experiments as the most effective in inducing resistance of wheat plants against S. nodorum (Yarullina et al., 2011). Then, sections of leaves of 7-day-old control and experimental wheat seedlings were placed in Petri dishes on damp cotton wool with the addition of benzimidazole (40 mg/L). Some of the leaves were infected with fungal spores by applying 4 μL of the suspension (105 spores/mL), according to the method (Veselova et al., 2021). Leaves inoculated with fungal spores in Petri dishes were placed in a thermostat for 24 hours and then transferred to a KBW E6 plant growth chamber (Binder GmbH, Germany).

Visual assessment of the degree of fungal development on wheat leaves. The development of the fungus S. nodorum on leaves was monitored daily. The area under the disease development curve in the variants was determined according to the method proposed by A.A. Marchenkova et al. (1991). Leaf lesion area was measured using ImageJ (https://imagej. nih.gov/ij/download.html).

RNA extraction and study of the relative gene expressions of the SnAGO genes. Isolation of total RNA from wheat leaves fixed in liquid nitrogen, as well as mycelium of the fungus S. nodorum SnB grown in vitro, was carried out using the Lyra reagent according to the protocol of Biolabmix (Russia, https://biolabmix.ru biolabmix.ru). The concentration of nucleic acids was measured using a Thermo Scientific™ NanoDrop™ 1000 spectrophotometer (ND1000WOC) at A260/A280. To synthesize cDNA, a reverse transcription reaction was performed using M-MuLV reverse transcriptase (Syntol, Russia). The nucleotide sequences of the studied SnAGO genes of the fungus S. nodorum were selected from the FunRNA database (http://funrna.riceblast.snu.ac.kr/, 04.12.2023). Primers for these genes were designed using the Internet programs “Primer-Blast” (https://www.ncbi.nlm.nih. gov/tools/primer-blast, 04.12.2023) and “PrimerQuest Tool” (https:/eu.idtdna.com/Primerquest, 04.12.2023) (the Table). To assess the level of gene transcription, we used the quantitative real-time PCR method on the CFX Connect Real-Time System device (Bio-Rad, USA). SYBR Blue reagent (Syntol, Russia) was used as an intercalating dye. The transcriptional activity of the fungal pathogen was assessed relative to the reference gene SnTUB, encoding the fungal tubulin protein (Fraaije et al., 2002). To assess the development of the fungus at the RNA level, we used an analysis of the ratio of transcripts of reference genes: the pathogen SnTub (Fraaije et al., 2002) and the host TaRLI, encoding a wheat RNase L inhibitor-like protein (Giménez et al., 2011) in experimental plants. According to the works of these authors, the expression of the noted genes of the fungus S. nodorum and wheat is not affected by environmental factors.

Bioinformatic and statistical analyzes. The experiments were carried out in triplicate. Mean values with standard errors (± SE) are shown in the figures. Statistical analysis of the obtained data was carried out in the Bio-Rad CFX Maestro 1.1 Version: 4.1.2433.1219 program (Bio-Rad, USA). Differences in the studied parameters between individual treatments were analyzed using analysis of variance. Alignment of nucleotide sequences and construction of a phylogenetic tree were carried out using the MEGA11: Molecular Evolutionary Genetics Analysis version 11.0.13 program (Tamura et al., 2021). The MUSCLE algorithm was used for alignment; phylogenetic trees were constructed using the Maximum Likelihood method (Tamura et al., 2021).

## Results

Based on previous studies, we selected bread wheat cultivars that contrast in their resistance to the fungus S. nodorum (Veselova et al., 2021). Previously, the effect of treating wheat seeds with SA and JA on the subsequent formation of resistance to the fungus S. nodorum in seedlings was also assessed (Yarullina et al., 2011). These works showed that SA and JA could significantly reduce the severity of the development of SNB in wheat

**Fig. 1.**
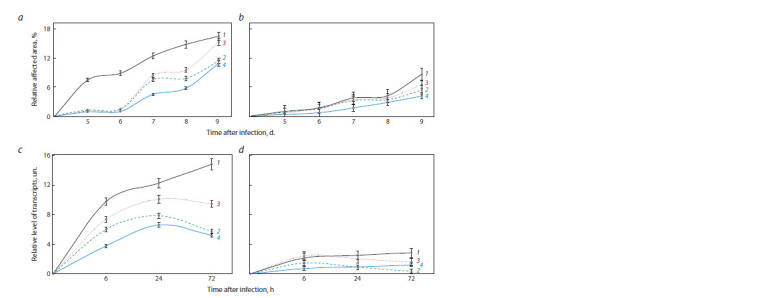
Comparative changes in leaf damage area (a, b) and the level of transcripts of the fungal reference SnTub gene in relation to the wheat TaRLI gene (c, d ) in the leaves of susceptible (a, c) and resistant (b, d ) cultivars of common wheat in the control (1) and after pre-treatment with salicylic (2), jasmonic (3) acids and their composition (4).

In this work, at the first stage, we analyzed the degree of fungal development in plant leaf tissues under normal conditions and under conditions of inducing the plant defense system using SA and JA pretreatment. The intensity of the formation of an infectious spot and the rapid development of necrosis in the leaves of the Zhnitsa cultivar showed a high degree of susceptibility of this cultivar to the pathogen strain we used (Fig. 1, a). The disease symptoms began to appear in the form of brown spots already on the 4th day after inoculation of the leaves, and on the 7th day after infection, the leaves were significantly affected. Under the same conditions, on the resistant cultivar Omskaya 35, SNB developed less intensively (see Fig. 1, b, d ). Accordingly, during the experiment, we confirmed the degree of susceptibility of the studied cultivars in terms of resistance to the fungus S. nodorum. In the variant of pre-treatment of seeds with SA and JA, as previously found (Yarullina et al., 2011), inhibition of the development of SNB on leaves infected with the fungus was observed, which suggests a systemic immunizing effect of these compounds. We found a particularly good protective effect in the variant of pre-sowing treatment of wheat seeds with a composition of SA and JA.

It is obvious that the successful development of the fungus S. nodorum in plant tissues is accompanied by the accumulation of its biomass and, accordingly, a change in the ratio of proteins and nucleic acids between the host and the pathogen. Based on this, we analyzed the ratio of transcript levels of reference genes: the pathogen SnTub and the host TaRLI in experimental plants. As can be seen from the data obtained (see Fig. 1, c, d ), the ratio of cDNA of the SnTub gene to the TaRLI gene in the susceptible Zhnitsa cultivar during the observed period was much higher than in the resistant one. This ratio noticeably decreased in plants pre-treated with SA and JA, as well as their composition (most noticeable after treatment of seeds with SA and JA).

In the resistant cultivars, the most pronounced decrease in the SnTub/TaRLI ratio was observed after treatment with SA. These data indicate the important contribution of the salicylate- induced pathway in the formation of resistance of wheat plants against the S. nodorum pathogen. Accordingly, we can say that the SnTub/TaRLI indicator is a convenient marker for early rapid diagnosis of resistance of wheat plants to the pathogenic fungus S. nodorum, as well as for assessing changes in this resistance at different stages of the formation of the relationship between the host plant and the specified pathogen

**Fig. 2.**
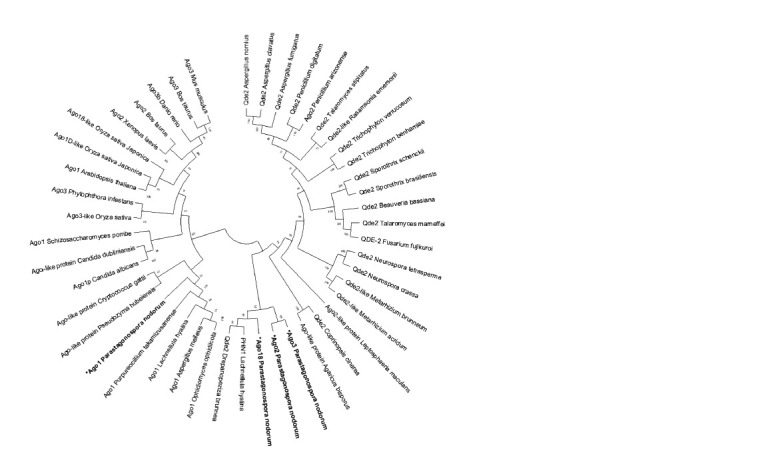
Phylogenetic tree of the AGO and QDE genes in various organisms. Sequences of fungi studied in this work are highlighted in bold.

To identify SnAGO genes encoding AGO family proteins in the S. nodorum genome, an analysis of the FunRNA database (Choi et al., 2014) was carried out using the annotated S. nodorum genome sequence (Hane et al., 2007). This approach allowed us to identify the SNOG_12157 locus. The phylogenetic tree of AGO genes is presented in Figure 2. As can be seen, the structure of the same AGO genes in different organisms has greater homology than the structure of different genes of a given family in representatives of the same genus/ taxon. Thus, the AGO1 gene we selected for analysis in the pathogen S. nodorum turned out to be on the same branch as the AGO1 genes of fungi from other genera or even divisions, and not with the AGO2, AGO3 and AGO18 genes of the same species. It is also noteworthy that, according to the obtained result, the Qde2 genes, being homologues of AGO1 (Jo et al., 2018), are located on a completely different phylogenetic branch of fungal genes. This classification of AGO1 into a separate group from AGO2 and AGO3 correlates with similar results obtained in other studies (Zhang et al., 2015; Ahmed et al., 2021).

Subsequent BlastP sequence analysis identified multiple genes putatively encoding fungal SnAGOs based on matches to known motifs characteristic of AGO genes. On this basis, the SnAGO genes were named SnAGO1, SnAGO2, SnAGO3, and SnAGO18, respectively, and were selected for further analysis of transcription activity. Primers for assessing the expression of the SnAGO1, SnAGO2, SnAGO3 and SnAGO18 genes are presented in the Table.

**Table 1.**
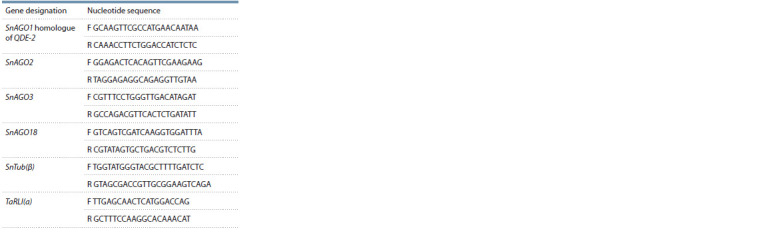
Primers of SnAGO gene loci of the fungus S. nodorum

**Fig. 3.**
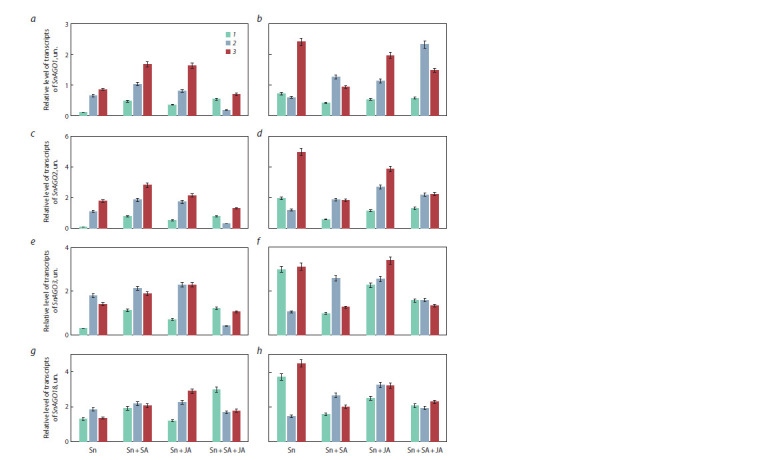
Changes in the level of transcripts of genes of the AGO family in the fungus S. nodorum in the leaves of susceptible (Zhnitsa) (a, c, e, g) and resistant (Omskaya 35) (b, d, f, h) wheat grown from salicylic (SA) and jasmonic (JA) acids treated seeds in 6 (1), 24 (2) and 72 (3) hours after infection. a, b, SnАGO1 (Snog_12157); c, d, SnАGO2 (Snog_10544); e, f, SnАGO3 (Snog_10546); g, h, SnАGO18 (Snog_12309).

Previously, it was not known how the transcriptional activity of fungal genes responsible for the formation of the RNAi phenomenon changes under conditions of infection of susceptible and resistant wheat cultivars. To do this, we analyzed the level of SnAGO gene transcripts identified in the fungus S. nodorum under infection conditions in bread wheat cultivars contrasting in resistance to this pathogen, pretreated with SA and JA, as well as with the SA + JA composite (Fig. 3). As can be seen, when wheat leaves are infected with fungal spores during the experiment, transcripts of the SnAGO1, SnAGO2 and SnAGO3 genes accumulate. Moreover, it is noteworthy that on the resistant cultivar Omskaya 35, the level of gene transcripts increases during the experiment.

Treatment of seeds with SA and JA, as well as their composition, was accompanied by locus-specific changes in the levels of transcripts of fungal SnAGO genes in a pathogenic system with both resistant and susceptible wheat cultivars (see Fig. 3). Most loci showed transcript accumulation; however, the extent of accumulation varied depending on the locus and the treatment. For example, the SnAGO1, SnAGO2 and SnAGO18 genes on the resistant cultivar Omskaya 35 as a result of seed treatment with JA were expressed to a greater extent than under the influence of SA. The composition of SA and JA reduced the activity of the SnAGO2 and SnAGO3 genes more pronouncedly in comparison with untreated and infected samples

In fungus-infected leaf tissues of a resistant cultivar, pretreated with both SA, JA, and their composition, after 24 hours of the experiment, the level of transcription of all studied genes (mostly SnAGO1) was higher compared to untreated plants after the same time of infection, but after 72 hours, it was lower than the corresponding levels in untreated plants (with the exception of SnAGO3 after JA treatment). In susceptible plants, throughout the experiment, the expression of the SnAGO1 and SnAGO3 genes increased approximately equally under the influence of both SA and JA; separately, the expression of the following genes increased to the greatest extent: SnAGO2 under the influence of SA and the SnAGO18 gene under the influence of JA, compared with control infected plants. When wheat seeds were treated with a mixture of SA and JA, the expression level of all SnAGO genes in the susceptible cultivar increased after 6 hours of infection (3-fold in the case of SnAGO18).

Thus, the obtained results showed that the SnAGO genes, encoding one of the key enzymes of the RNAi mechanism, in fungal cells, when interacting with plant cells, respond to the degree of host resistance and increase their transcriptional activity in resistant cultivars. Accordingly, the higher level of SnAGO transcripts in pathogen-infected wheat leaves confirms previous suggestions about the important role of this group of proteins in the formation of compatible relationships between wheat and the fungus S. nodorum (Shen et al., 2014).

**Fig. 4.**
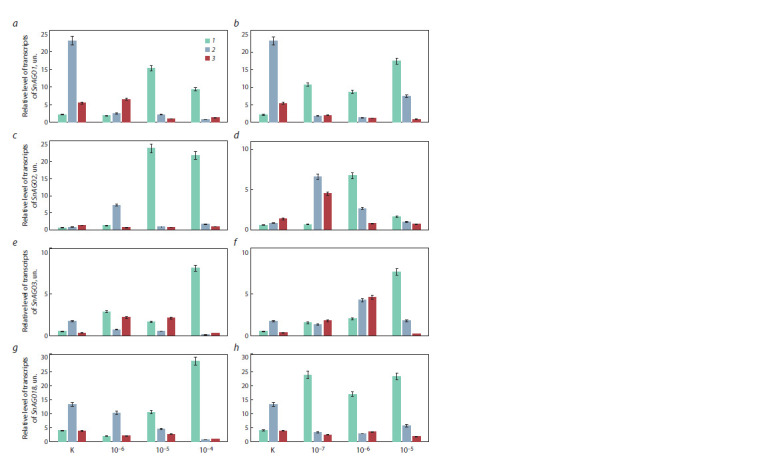
Changes in the level of transcripts of the SnAGO genes of S. nodorum grown on a liquid nutrient medium with the addition of salicylic (SA) (a, c, e, g) and jasmonic (JA) (b, d, f, h) acids of various concentrations (M) in 5 (1), 7 (2) and 14 (3) days after planting. a, b, SnАGO1 (Snog_12157); c, d, SnАGO2 (Snog_10544); e, f, SnАGO3 (Snog_10546); g, h, SnАGO18 (Snog_12309).

From our point of view, it is interesting to evaluate the direct response of the fungal genome to the effects of SA and JA on a nutrient medium. Thus, using mycelium of the fungus S. nodorum grown in liquid culture, we assessed the expression status of SnAGO genes when SA and JA solutions of various concentrations were added to the medium on days 5, 7, and 14 after planting on the medium (Fig. 4). It was found that when the fungus was cultivated on a nutrient medium, the activity of the SnAGO1 gene in control samples increased on the 7th day after the start of cultivation. The activity of the SnAGO2 gene in control colonies of the fungus increased during the experiment, but the effect was less pronounced. When SA was added to the nutrient medium, a pronounced increase in the level of transcripts of all the studied SnAGO genes was observed already on the 5th day, and the degree of accumulation of transcripts of these genes was directly proportional to the concentration of the added substance. Similar data were obtained when JA was added to the medium. At the same time, it is noteworthy that the addition of signaling molecules at all JA concentrations and relatively high SA concentrations shifts the accumulation of transcripts to the earliest period – 5 days. Particular attention should be paid to the fact that the SnAGO1 and SnAGO18 genes turned out to be the most sensitive to the addition of JA to the nutrient medium.

## Discussion

Assessing the mechanisms of formation of plant resistance, especially against pathogens, is an urgent task, the solution of which will make it possible to effectively regulate it and achieve higher productive properties. The diversity of feeding methods of phytopathogenic fungi contributed to the evolutionary formation of various ways of protecting plants from them (McCombe et al., 2022). For example, against biotrophic pathogens that feed on living plant tissues, defense mechanisms are launched, where the SA-dependent signaling pathway plays an active role, forming systemic acquired resistance. JA triggers another signaling pathway, called systemic induced resistance, which protects plants from necrotrophic pathogens and insects. Numerous studies have shown that the causative agent of SNB is characterized by a hemibiotrophic mode of nutrition on wheat plants, combining a biotrophic phase of development at the very beginning of pathogenesis with a subsequent necrotrophic one; however, it is also capable of saprotrophic growth on nutrient media (Oliver et al., 2012). Accordingly, depending on the timing of pathogen development in plant tissues, host defense systems must clearly recognize these transition stages.

We have previously shown that the highly virulent strain of the pathogenic fungus S. nodorum SnB can overcome the defense of wheat associated with the pro-/antioxidant system, including the activation of catalases (Troshina et al., 2010) and chitin deacetylases (Maksimov et al., 2011), and also the accumulation of various effector molecules (Veselova et al., 2021). In this work, we analyzed a number of S. nodorum genes encoding SnAGO nuclease proteins involved in the RNAi mechanism, using it as a model object.

The RNAi phenomenon represents a unique and ancient mechanism for protecting the genome of eukaryotes, including fungi, from foreign genetic information, and also serves to regulate physiological processes. The most important key components of this complex immune mechanism are AGO RNA nucleases, which have been functionally characterized in model organisms (Choi et al., 2014). It has been shown that the RNAi mechanism in the eukaryotic immune system during the interaction of hosts with their parasites is a “double-edged instrument”, which, on the one hand, protects the host from the pathogen, and on the other hand, helps the pathogen disable the accumulation of the most essential key protective proteins of the host, in order to use plant resources for its own functioning. However, to date, the mechanisms of RNAi operation in fungal systems, especially during the development of diseases, have not been sufficiently studied, which limits our understanding of this mechanism

For example, the formation of compatibility between the host and the pathogen with the participation of plant proteins AGO1 of Brassica napus was shown under conditions of plant infection by the fungi V. dahliae and V. longisporum (Shen et al., 2014). In tomato and Arabidopsis plants, microRNAs secreted by the fungus Botrytis cinerea used the host AGO1 proteins to selectively suppress the translation of host mitogenactivated protein kinases (MAPKs), peroxiredoxin, and cell wall-associated kinase, and suppression of the accumulation of this protein in mutant Arabidopsis plants resulted in reduced susceptibility to the fungus (Weiberg et al., 2013). F. Dunker and co-workers (2020) showed on Arabidopsis plants that similar recruitment of the host protein complex AGO1 is also characteristic of the oomycete Hyaloperonospora arabidopsidis, a pathogen phylogenetically distant from fungi, suggesting a property common to pathogens

An important role of the RNAi mechanism in the growth and development of fungi has been revealed, which is naturally reflected in their ability to infect plants. Thus, Δago1 mutants of the fungus Colletotrichum higginsianum showed severe defects in conidial morphology (Campo et al., 2016). Deletion of the FgAGO2 gene of the fungus Fusarium graminearum did not affect the fungal phenotype during the asexual phase (Chen et al., 2015), but this gene was found to be important during the fungal sporulation and ascospore maturation stages (Zeng et al., 2018). In another species of fungus, Fusarium oxysporum f. sp. lycopersici strain 4287, mutants with suppressed expression of the FoQDE-2 (AGO1) gene (Jo et al., 2018) exhibited reduced virulence against tomato plants.

At the moment, there are practically no works that would discuss the behavior of fungal pathogen genes encoding proteins responsible for RNAi under conditions of direct exposure to biostimulants and resistance inducers, for example, SA and JA, or indirect exposure through plant infection. There is conflicting information about the role of plant resistance inducers in the operation of the RNAi mechanism in plant tissues under the direct influence of SA and JA and in response to infection by pathogens. For example, some authors show that SA-induced pathogen resistance affects plant RNAi mechanisms only through the host RNA-dependent RNA polymerase (RDR1) and is coordinated by this protein (Lee et al., 2016). At the same time, there is information that in plants, for example, rice, the expression levels of the OsAGO1a, OsAGO2 and OsAGO18 genes turned out to be associated with JA, and in mutant rice plants coi1-13, unable to transmit JA signals, they were lower than in wild plants (Yang et al., 2020). This is especially interesting due to the fact that in the pathogenic wheat fungus S. nodorum, the role of RNAi in pathogenicity still remains unresolved. At the same time, the participation of RNAi in the development of pathogenicity of Zymoseptoria tritici, another fungus that causes septoria leaf blight on wheat, was recently analyzed after knockout of the AGO1 and AGO2 genes in its genome (Kettles et al., 2019; Ma et al., 2020). The analysis did not reveal qualitative phenotypic changes in the development of SNB symptoms on the susceptible wheat cultivar Bobwhite. Fungal strains mutant for the AGO genes did not lose their virulence properties against wheat plants. From the results obtained, the authors conclude that these proteins do not play a significant role in the development of SNB. At the same time, analysis of the expression status of genes responsible for the operation of the RNAi mechanism in the pathogenic fungus V. nonalfalfae, which causes verticillium wilt in hops (Humulus lupulus L.), showed that more virulent strains of the pathogen have a higher level of accumulation of VnAGO transcripts (Jeseničnik et al., 2019).

We showed that in the pathogen-resistant to S. nodorum wheat cultivar Omskaya 35, the accumulation of transcripts of the studied SnAGO genes during fungal pathogenesis in leaf tissues was more active than in the susceptible cultivar, which suggests the involvement of the studied genes in the process of overcoming the host’s defense system and the possibility of regulating activity of these genes depending on the degree of host resistance. These assumptions are further confirmed in experimental variants where plants were immunized by pre-sowing seed treatment with SA and JA, as well as their composition. As can be seen, the level of transcripts of pathogenic SnAGO genes in these variants also turns out to be higher than in the control ones, although, judging by the ratio of housekeeping genes SnTub/TaRLI, the content of the SnTub gene in plants immunized with SA and JA is significantly lower

We obtained interesting results when assessing the transcriptional activity of SnAGO genes after direct exposure of the fungal mycelium in culture to SA and JA. High concentrations of SA enhanced the accumulation of transcripts of the studied SnAGO genes at earlier times of cultivation, while low concentrations did not produce this effect. JA stimulated the accumulation of SnAGO1, SnAGO3, SnAGO18 transcripts at early stages of cultivation and at lower concentrations compared to SA. It can be assumed that fungal SnAGO genes are sensitive to the direct effects on the fungal mycelium of these signaling molecules, which are positioned as inducers of plant defense systems against pathogens. Thus, we have shown that the fungal RNAi system actively responds to the addition of SA and JA to the cultivation medium, and also participates in the process of plant infection. At the same time, artificial stimulation of the protective properties of the plant also triggers the accumulation of SnAGO transcripts of the pathogen. Our data suggest that the AGO genes involved in the RNAi system of the fungus S. nodorum strain SnB interact effectively with the infecting plant and, most likely, this interaction depends on the degree of host resistance.

The possibility of assessing the degree of development of pathogens in plant tissues using molecular biological methods that assess the level of accumulation of pathogen nucleic acids in tissues is very important, since visual assessment of disease symptoms is often subjective and influenced by various factors. This work uses the SnTub/TaRLI method for assessing the accumulation of pathogen genetic material in plant tissues by comparing the ratio of transcript levels of fungal and wheat housekeeping genes. Estimation of the SnTub/TaRLI transcript ratio correlated with the results of visual observation of disease development on leaves. The results also showed that the most effective in inducing the protective properties of wheat plants against SNB was pre-sowing treatment of seeds with JA, as well as with a composition of JA and SA (see Fig. 1). Using the level of the SnTub/TaRLI ratio, we confirmed the dynamics of accumulation of biological material of the pathogenic fungus, but at earlier periods of observation. In addition, we demonstrated the possibility of using the data obtained on the transcriptional activity of the AGO genes of the fungus S. nodorum strain SnB as an objective indicator of the activation of its RNAi system.

## Conflict of interest

The authors declare no conflict of interest.

## References

Ahmed F.F., Hossen M.I., Sarkar M.A.R., Konak J.N., Zohra F.T.,
Shoyeb M., Mondal S. Genome-wide identification of DCL, AGO
and RDR gene families and their associated functional regulatory
elements analyses in banana (Musa acuminata). PLoS One. 2021;
16(9):e0256873. DOI 10.1371/journal.pone.0256873

Campo S., Gilbert K.B., Carrington J.C. Small RNA-based antiviral
defense in the phytopathogenic fungus Colletotrichum higginsianum.
PLoS Pathog. 2016;12:e1005640. DOI 10.1371/journal.ppat.
1005640

Chen Y., Gao Q., Huang M., Liu Y., Liu Z., Liu X., Ma Z. Characterization
of RNA silencing components in the plant pathogenic fungus
Fusarium graminearum. Sci. Rep. 2015;5:12500. DOI 10.1038/
srep12500

Choi J., Kim T., Jeon J., Wu J., Song H., Asiegbu F.O., Lee Y.H. fun-
RNA: a fungi-centered genomics platform for genes encoding key
components of RNAi. BMC Genomics. 2014;15(Suppl. 9):S14. DOI
10.1186/1471-2164-15-S9-S14

Dunker F., Trutzenberg A., Rothenpieler J.S., Kuhn S., Pröls R., Schreiber
T., Tissier A., Kemen A., Kemen E., Hückelhoven R., Weiberg
A. Oomycete small RNAs bind to the plant RNA-induced silencing
complex for virulence. Elife. 2020;9:e56096. DOI 10.7554/
eLife.56096

Giménez M.J., Pistón F., Atienza S.G. Identification of suitable reference
genes for normalization of qPCR data in comparative transcriptomics
analyses in the Triticeae. Planta. 2011;233(1):163-173.
DOI 10.1007/s00425-010-1290-y

Feng H., Xu M., Liu Y., Gao X., Yin Z., Voegele R.T., Huang L. The
distinct roles of Argonaute protein 2 in the growth, stress responses
and pathogenicity of the apple tree canker pathogen. Forest Pathol.
2017;47(5):e12354. DOI 10.1111/efp.12354

Fraaije B.A., Lovel D.J., Baldwin S. Septoria epidemics on wheat:
combined use of visual assessment and PCR-based diagnostics of
identity mechanism of diseases escape. Plant Protect. Sci. 2002;
38(11):421-424. DOI 10.17221/10512-PPS

Hane J.K., Lowe R.G., Solomon P.S., Tan K.C., Schoch C.L., Spatafora
J.W., Crous P.W., Kodira C., Birren B.W., Galagan J.E., Torriani
S.F., McDonald B.A., Oliver R.P. Dothideomycete plant interactions
illuminated by genome sequencing and EST analysis of the
wheat pathogen Stagonospora nodorum. Plant Cell. 2007;19(11):
3347-3368. DOI 10.1105/tpc.107.052829

Jeseničnik T., Štajner N., Radišek S., Jakše J. RNA interference core
components identified and characterized in Verticillium nonalfalfae,
a vascular wilt pathogenic plant fungi of hops. Sci. Rep. 2019;
9(1):8651. DOI 10.1038/s41598-019-44494-8

Jo S.M., Ayukawa Y., Yun S.H., Komatsu K., Arie T. A putative RNA
silencing component protein FoQde-2 is involved in virulence of the
tomato wilt fungus Fusarium oxysporum f. sp. lycopersici. J. Gen.
Plant Pathol. 2018;84:395-398. DOI 10.1007/s10327-018-0800-9

Kettles G.J., Hofinger B.J., Hu P., Bayon C., Rudd J.J., Balmer D.,
Courbot M., Hammond-Kosack K.E., Scalliet G., Kanyuka K.
sRNA profiling combined with gene function analysis reveals a lack
of evidence for cross-kingdom RNAi in the wheat – Zymoseptoria
tritici pathosystem. Front. Plant Sci. 2019;10:892. DOI 10.3389/
fpls.2019.00892

Lee W.S., Fu S.F., Li Z., Murphy A.M., Dobson E.A., Garland L.,
Chaluvadi S.R., Lewsey M.G., Nelson R.S., Carr J.P. Salicylic acid
treatment and expression of an RNA-dependent RNA polymerase 1
transgene inhibit lethal symptoms and meristem invasion during tobacco
mosaic virus infection in Nicotiana benthamiana. BMC Plant
Biol. 2016;16:15. DOI 10.1186/s12870-016-0705-8

Ma X., Wiedmer J., Palma-Guerrero J. Small RNA bidirectional crosstalk
during the interaction between wheat and Zymoseptoria tritici.
Front. Plant Sci. 2020;10:1669. DOI 10.3389/fpls.2019.01669

Maksimov I.V., Valeev A.Sh., Safin R.F. Acetylation degree of chitin
in the protective response of wheat plants. Biochemistry (Moscow).
2011;76(12):1342-1346. DOI 10.1134/S0006297911120078

Marchenkova A.A., Nettevich E.D., Tushinsky T.Yu. Resistance of
spring wheat to septoria blight. Vestnik Selskokhozyaystvennoy
Nauki
= Herald of Agricultural Sciences. 1991;7:110-115 (in Russian)

Meng H., Wang Z., Wang Y., Zhu H., Huang B. Dicer and Argonaute
genes involved in RNA interference in the entomopathogenic fungus
Metarhizium robertsii. Appl. Environ. Microbiol. 2017;83(7):
e03230-16. DOI 10.1128/AEM.03230-16

McCombe C.L., Greenwood J.R., Solomon P.S., Williams S.J. Molecular
plant immunity against biotrophic, hemibiotrophic, and necrotrophic
fungi. Essays Biochem. 2022;66(5):581-593. DOI 10.1042/
EBC20210073

Neupane A., Feng C., Mochama P.K., Saleem H., Lee Marzano S.Y.
Roles of Argonautes and Dicers on Sclerotinia sclerotiorum antiviral
RNA silencing. Front. Plant Sci. 2019;10:976. DOI 10.3389/fpls.
2019.00976

Oliver R.P., Friesen T.L., Faris J.D., Solomon P.S. Stagonospora nodorum:
From pathology to genomics and host resistance. Annu. Rev.
Phytopathol. 2012;50:23-43. DOI 10.1146/annurev-phyto-081211-
173019

Pradhan M., Pandey P., Baldwin I.T., Pandey S.P. Argonaute 4 modulates
resistance to Fusarium brachygibbosum infection by regulating
Jasmonic acid signaling. Plant Physiol. 2020;184(2):1128-1152.
DOI 10.1104/pp.20.00171

Raman V., Simon S.A., Demirci F., Nakano M., Meyers B.C., Donofrio
N.M. Small RNA functions are required for growth and development of Magnaporthe oryzae. Mol. Plant Microbe Interact.
2017;30(7):517-530. DOI 10.1094/MPMI-11-16-0236-R

Shein M.Yu., Burkhanova G.F., Merzlyakova A.Yu., Maksimov I.V.
Changes in transcriptional activity of TaAGO2 and TaAGO4 genes
in wheat plants at infection with Stagonospora nodorum Berk. Trudy
Kubanskogo Gosudarstvennogo Agrarnogo Universiteta = Works
of the Kuban State Agrarian University. 2021;5(92):196-200. DOI
10.21515/1999-1703-92-196-200 (in Russian)

Shen D., Suhrkamp I., Wang Y., Liu S., Menkhaus J., Verreet J.A.,
Fan L., Cai D. Identification and characterization of microRNAs in
oilseed rape (Brassica napus) responsive to infection with the pathogenic
fungus Verticillium longisporum using Brassica AA (Brassica
rapa) and CC (Brassica oleracea) as refer. New Phytol. 2014;
204(3):577-594. DOI 10.1111/nph.12934

Tamura K., Stecher G., Kumar S. MEGA 11: Molecular evolutionary
genetics analysis version 11. Mol. Biol. Evol. 2021;38(7):3022-
3027. DOI 10.1093/molbev/msab120

Troshina N.B., Surina O.B., Cherepanova E.A., Yarullina L.G., Maksimov
I.V. Comparative evaluation of H2O2-degrading activity
of aggressive Septoria nodorum strains. Mikologiya i Fitopatologiya
= Mycology and Phytopathology. 2010;44(3):273-279 (in Russian)

Veselova S., Nuzhnaya T., Burkhanova G., Rumyantsev S., Maksimov
I. Reactive oxygen species in host plant are required for an
early defense response against attack of Stagonospora nodorum
Berk. necrotrophic effectors SnTox. Plants. 2021;10(8):1586. DOI
10.3390/plants10081586

Wang Q., An B., Hou X., Guo Y., Luo H., He C. Dicer-like proteins regulate
the growth, conidiation, and pathogenicity of Colletotrichum
gloeosporioides from Hevea brasiliensis. Front Microbiol. 2018;8:
2621. DOI 10.3389/fmicb.2017.02621

Weiberg A., Wang M., Lin F.M., Zhao H., Zhang Z., Kaloshian I.,
Huang H.D., Jin H. Fungal small RNAs suppress plant immunity
by hijacking host RNA interference pathways. Science. 2013;
342(6154):118-123. DOI 10.1126/science.1239705

Yang Z., Huang Y., Yang J., Yao S., Zhao K., Wang D., Qin Q., Bian Z.,
Li Y., Lan Y., Zhou T., Wang H., Liu Ch., Wang W., Qi Y., Xu Z.,
Li Y. Jasmonate signaling enhances RNA silencing and antiviral
defense in rice. Cell Host Microbe. 2020;28(1):89 103.e8. DOI
10.1016/j.chom.2020.05.001

Yarullina L.G., Troshina N.B., Cherepanova E.A., Zaikina E.A.,
Maksimov I.V. Salicylic and Jasmonic acids in regulation of the
proantioxidant state in wheat leaves infected by Septoria nodorum
Berk. Prikladnaya Biokhimiya i Mikrobiologiya = Applied Biochemistry
and Microbiology. 2011;47:549-555. DOI 10.1134/S000368
3811050176 (in Russian)

Zhang H., Xia R., Meyers B.C., Walbot V. Evolution, functions, and
mysteries of plant ARGONAUTE proteins. Curr. Opin. Plant Biol.
2015;27:84-90. DOI 10.1016/j.pbi.2015.06.011

Zeng W., Wang J., Wang Y., Lin J., Fu Y., Xie J., Jiang D., Chen T.,
Liu H., Cheng J. Dicer-Like proteins regulate sexual development
via the biogenesis of perithecium-specific MicroRNAs in a plant
pathogenic fungus Fusarium graminearum. Front Microbiol. 2018;
9:818. DOI 10.3389/fmicb.2018.00818

